# The Impact of Blood Morphological Parameters on Treatment Outcomes in Tennis Elbow Patients Receiving Platelet-Rich Plasma (PRP) Therapy: A Prospective Study

**DOI:** 10.3390/jcm13010077

**Published:** 2023-12-22

**Authors:** Karol Szyluk, Rostyslav Bubnov, Alicja Jarosz, Rafał Reguła, Piotr Grabowski, Joanna Iwanicka, Tomasz Iwanicki, Marcin Gierek, Dominik Sieroń, Andreas Christe, Paweł Niemiec

**Affiliations:** 1 Department of Physiotherapy, Faculty of Health Sciences in Katowice, Medical University of Silesia in Katowice, Medyków 18 Str., 40-752 Katowice, Poland; 2 District Hospital of Orthopaedics and Trauma Surgery, Bytomska 62 Str., 41-940 Piekary Śląskie, Poland; r.regula@urazowka.piekary.pl (R.R.); p.grabowski@urazowka.piekary.pl (P.G.); 3 Clinical Hospital “Pheophania” of State Affairs Department, Zabolotny Institute of Microbiology and Virology, National Academy of Sciences of Ukraine, 03143 Kyiv, Ukraine; 4 Department of Biochemistry and Medical Genetics, School of Health Sciences in Katowice, Medical University of Silesia in Katowice, Medyków 18 Str., 40-752 Katowice, Poland; alicja.jarosz@sum.edu.pl (A.J.); jiwanicka@sum.edu.pl (J.I.); tiwanicki@sum.edu.pl (T.I.); pniemiec@sum.edu.pl (P.N.); 5 Center for Burns Treatment, Jana Pawła II Str., 41-100 Siemianowice Śląskie, Poland; marcin.gierek@clo.com.pl; 6 Department of Radiology SLS, Inselgroup, Bern University Hospital, University of Bern, Freiburgstrasse 10, 3010 Bern, Switzerland; dominik.sieron.ch@gmail.com (D.S.); andreas.christe@insel.ch (A.C.); 7 Department of Diagnostic, Interventional and Pediatric Radiology, Inselspital, Bern University Hospital, University of Bern, Freiburgstrasse 10, 3010 Bern, Switzerland

**Keywords:** tennis elbow, lateral elbow tendinopathy, lateral epicondylitis, platelet-rich plasma (PRP), elbow pain

## Abstract

Platelet-rich plasma (PRP) therapy holds substantial promise for the treatment of tennis elbow, a complex and challenging musculoskeletal condition. The aim of the study was to assess whether there are correlations between the levels of individual morphotic elements determined in whole blood and the outcomes of tennis elbow treatment with PRP injection, as measured using patient-reported outcome measures (PROMs) such as the Visual Analog Scale (VAS), Quick Disabilities of the Arm, Shoulder, and Hand (QDASH), and Patient-Rated Tennis Elbow Evaluation (PRTEE). A prospective analysis was conducted on 107 patients (132 elbows) undergoing lateral epicondylitis treatment with (PRP) injections. Patients completed VAS, PRTEE, and QDASH questionnaires on the day of PRP administration and at established checkpoints (2, 4, 8, 12, 24, 52, and 104 weeks). Minimal clinically important difference (MCID) was employed to assess the treatment effects. Then, correlations were measured within each PROM, and the impact of the concentration of individual blood parameters on the MCID outcomes was assessed. Analyzing the relationships between the MCID+ and MCID− groups, significant correlations for the VAS and QDASH scales were observed. The level of individual morphotic elements in the blood may have influenced the treatment outcome, as measured using specific patient-reported outcome measures (PROMs). Regarding the VAS scale, factors favoring a positive treatment outcome included higher values of eosinophils (EOS) and basophils (BASO). For the QDASH scale, these factors were a lower value of mean corpuscular volume (MCV) and a higher mean corpuscular hemoglobin (MCH). The levels of certain blood parameters, such as EOS and BASO, in the current study influenced the classification of patients into MCID+ or MCID− groups, based on the VAS and QDASH scales.

## 1. Introduction

The term “tennis elbow” is commonly used to refer to lateral elbow tendinopathy (LET), also historically known as lateral epicondylitis. The latter term suggests an inflammatory etiology, and an alternative historical term is “Epicondylalgia” [1,2,3,4]. The diverse terms used reflect the evolving understanding of LET’s etiology over time. The recent literature, backed by histological studies, suggests a crucial role of overuse/trauma and degenerative processes [3,4,5]. In the development of tendinopathy, there is hyperplasia and hypertrophy of cells, changes in the shape of the tenocytes, a reduction in the nucleus-to-cytoplasm ratio, activation of fibroblasts producing collagen type 3, and hyperplasia of blood vessels, leading to the formation of a partially inadequate vascular bed [3,4,6,7,8]. LET is among the most common dysfunctions of the elbow joint, marked by pain in the lateral epicondyle region of the humerus, radiating proximally and distally, along with a decrease in muscle strength. It affects approximately 3% of the population, most commonly between the age of 30 and 65 years, with equal frequency in both men and women [6,9,10]. Characteristic to LET is its self-limiting nature, with 80–90% of patients reporting symptom relief within a year of onset [8,9,10,11,12,13,14,15,16,17,18,19,20,21,22,23,24,25]. There is evidence showing the benefit of PRP for the treatment of knee osteoarthritis, various tendinopathies, acute soft tissue injuries, alopecia, and other dermatological disorders [11,12,13,14]. Among many different tennis elbow therapies, platelet-rich plasma (PRP) injections are increasingly applied [11,12,13,14,16,17,18,19,20,21]. There are many recent studies that show clear benefits for the patients regarding the use of PRP, but there is still a lack of identified factors influencing the selection of patients susceptible to treatment [11,12,14,16,17,18,19,20]. Although a correlation between gene polymorphism and treatment outcomes has been noted, it is not commonly used in the selection of patients for PRP administration [16,17,18,19,20].The authors highlight the importance of seeking simpler methods to identify patients amenable to PRP therapy. Therefore, they aim to explore the relationship between the treatment outcomes and specific morphotic parameters in the peripheral blood. Additionally, identifying these relationships may contribute to further research on the mechanism of treating LET. The aim of the current study is to assess whether there are correlations between the levels of specific morphotic elements in the whole blood and the outcome of tennis elbow treatment using PRP injections, as measured using common patient-reported outcome measures (PROMs) such as the Visual Analog Scale (VAS), Quick Disabilities of the Arm, Shoulder, and Hand (QDASH), and Patient-Rated Tennis Elbow Evaluation (PRTEE).

## 2. Materials and Methods

### 2.1. Design and Ethics

The current research had a prospective design and was conducted in accordance with the standards of Polish and international law, including the Helsinki Declaration from 1975 and all its updates up to the project’s commencement date. The research project was approved by the bioethical committee of the Medical University of Silesia in Katowice, Poland (KNW/0022/KB1/24/I/17). The study included 107 patients (132 elbows) undergoing treatment for LET with the use of platelet-rich plasma (PRP) injections. In the study group, there were 65 women (77 elbows) and 42 men (55 elbows), with an age range from 24 to 64 years (median = 46.00  ±  5.50). 

All patients included in the study were treated at two centers: the 6th Department of Orthopedics and Traumatology of the Musculoskeletal System at the Provincial Hospital in Piekary Śląskie, Poland, and the Department of Orthopedics and Traumatology of the Musculoskeletal System in Jaworzno, Poland. Patient inclusion, as well as the PRP injection, was performed by two orthopedic specialists (K.S.—Piekary Śląskie and W.K.—Jaworzno), each with extensive experience, in accordance with the approved research protocol of the ethics committee. The observation period lasted for 104 weeks. On the day of PRP administration and at set checkpoints (2, 4, 8, 12, 24, 52, and 104 weeks post-PRP injection), patients completed the VAS, PRTEE, and QDASH questionnaires. Throughout the study, data were also collected on factors that could affect the questionnaire results, such as medications taken, implemented physiotherapy, or surgery due to lateral elbow tendinopathy.

### 2.2. Platelet-Rich Plasma (PRP) Administration Procedure and Patient Blood Sample Analysis

On the day of PRP administration, patients underwent a full blood draw for subsequent laboratory analysis. For PRP separation, we consistently used the Arthrex Autologous Conditioned Plasma (ACP) system (Arthrex GmbH, Munich, Germany). From each patient, 12 mL of whole blood was drawn using a 1.2 mm diameter needle, and the blood was mixed with 3.13% sodium citrate (MediPac^®^ GmbH, Königswinter, Germany). To obtain PRP, the whole blood underwent centrifugation using the ROTOFIX 32 A centrifuge (Andreas Hettich GmbH & Co., Tuttlingen, Germany). The full blood was centrifuged for 5 min at a speed of 1500 rpm. Using this described procedure, approximately 2.5 to 3.5 mL of PRP was obtained, promptly administered to the patient as an injection to the soft tissues surrounding the common extensor tendon at the lateral epicondyle. All injections were administered in a facility meeting hospital sanitary requirements, maintaining a consistent temperature and light exposure throughout the procedure.

### 2.3. Patient Selection

The patients included in the study were diagnosed with tennis elbow based on:

Positive anamnesis: Elbow pain in the region of the extensor muscle attachment (spontaneous or provoked by exertion), muscle strength weakness, and reported prolonged overuse/microtrauma associated with occupational or sports-related activities. In the cohort of all patients enrolled in the study, the symptoms exhibited a chronic nature, with their onset occurring at least 3 months prior to the administration of the injection.

Positive physical examination: Patients were included in the study based on a positive outcome in the Thomson, Mills, or Cozen tests, along with identified palpation tenderness in the area of the lateral epicondyle of the humerus.

Additionally, only patients who had undergone the panel of laboratory tests (see Table 1) directly before the injection were included. The panel tested in each patient is indicated in Table 1 below.

Additionally the MPV/PLT ratio was also calculated.

Patients with complete patient-reported outcome measure (PROM) results at all follow-up time points were included in the study. All patients included in the study provided voluntary consent to participate.

The criteria for exclusion from the study included:

Previous injuries within the limb that could have affected its function.

Comorbidities (golfer’s elbow, rheumatoid arthritis, active cancer, cervical radiculopathy), pregnancy, previous surgical treatment for lateral elbow tendinopathy, drug use (steroids, non-steroidal anti-inflammatory drugs). Incomplete PROM results or mixed response during the follow-up (improvement and worsening) that could not be attributed to MCID positivity or negativity (see below).

Patient’s lack of co-operation, patients who did not complete the follow-up after signing voluntary consent to participate.

Refusal to participate in the study, patients who did not sign voluntary consent to participate in the study before the administration of PRP.

It is worth noting that the analysis excluded only those patients who underwent other forms of therapy after receiving PRP (regardless of the treatment outcomes). Those who had to use other forms of therapy, including NSAIDs before the injection, were not excluded. An exception was made for patients who received steroid injections within the 6 months preceding PRP administration; these patients were not included in the study. The exclusion of patients who underwent other forms of therapy (NSAIDs, physiotherapy, etc.) after PRP injection ensures that the results reported in this study reflect the natural course and response to the investigated parameters without the influence of additional treatments introducing confounding factors.

#### Minimal Clinically Important Difference (MCID)

To evaluate the treatment outcomes, commonly used patient-reported outcome measures (PROMs) were employed, specifically the Visual Analog Scale (VAS), Quick Version of Disabilities of the Arm, Shoulder, and Hand score (QDASH), and Patient-Rated Tennis Elbow Evaluation (PRTEE) questionnaires. The scoring ranges for the questionnaires were as follows: 0–10 for the VAS, where 0 corresponds to minimal pain and 10 to maximal pain, and 0–100 for QDASH and PRTEE, where 0 indicates minimal and 100 indicates maximal disability/pain. Significant and perceptible improvement in the patient’s health after therapy was defined with the achievement of the minimal clinically important difference (MCID) for a given PROM. The patient was considered to have achieved MCID when, during a specific observation period (weeks 2, 4, 8, 12, 24, 52, and 104 post-PRP injection), there was a reduction in the questionnaire score relative to the pre-therapy value by 1.5 points for the VAS, 15.8 points for QDASH, and 11 points for PRTEE [21,22,23].

MCID analysis was conducted at each follow-up point. Subsequently, each patient was assigned a sequence of results: improvement (MCID+) or lack of improvement (failure to reach the MCID threshold, MCID−). Based on the collected result series for each patient, two groups of patients were delineated for each individual PROM, which were subjected to further analysis:

The MCID+ group included patients with a positive treatment outcome, where improvement (achieving the MCID threshold) occurred at 2 or 4 weeks of observation and remained consistently up to the 104th week of observation,

The MCID− group consisted of patients with a poor treatment outcome, where, from the beginning to the end of the observation period, the MCID threshold was not achieved at any of the observation points.

Patients whose result series did not match the above profiles were excluded from further analysis.

Because three different scales with varying structures and question ranges were used in the study, the number of patients who met the inclusion criteria for each scale was different and amounted to 36 patients (46 elbows) for the VAS scale, 35 patients (47 elbows) for the QDASH scale, and 35 patients (49 elbows) for the PRTEE scale, respectively.

**Figure 1:**
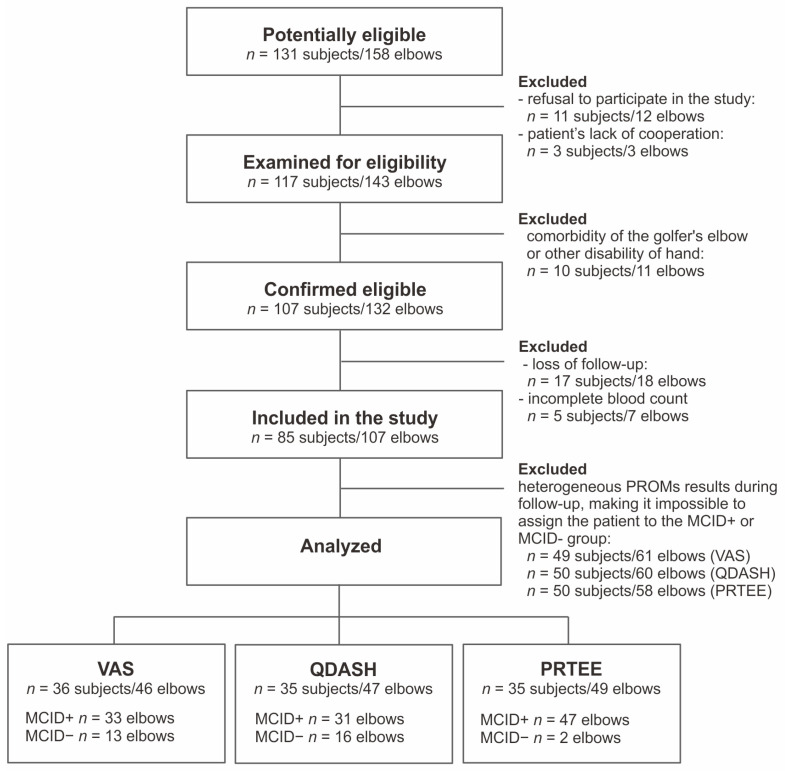
Flowchart of the study selection.

The flow diagram of the patients included in the study is presented in Figure 1.

In the initial stage of the analysis, the presence of a relationship between reaching the MCID threshold for each follow-up point and each individual PROM was examined, along with the results of the blood test. Subsequently, correlations between the outcomes of individual PROMs and blood tests at successive checkpoints were examined for all patients within the study group, without division into the MCID+ and MCID− groups.

In the subsequent stage of the analysis, differences between the MCID+ and MCID− groups were examined, taking into account individual parameters identified in the peripheral blood as well as the MPV-to-PLT ratio to assess their utility in predicting the treatment outcomes and identifying patients with a potential good response to the applied therapy.

Furthermore, predictability for the MCID+ or MCID− classification based on the levels of the blood parameters was measured.

### 2.4. Statistical Analysis

Microsoft Excel software version 2002 (Microsoft Corporation, Redmond, WA, USA) was used to store the data. Statistical analysis was performed using the Statistica 13.0 software (TIBCO Software Inc., Palo Alto, CA, USA). The Shapiro–Wilk test was used to check the normality of the distribution. Since the quantitative variables did not follow a normal distribution, data were compared using the Mann–Whitney U test. To assess the strength of the relationship between the MCID test results, Spearman’s rank correlation (Spearman’s rho) was utilized. In the case of Spearman’s rho correlation, the results were first ranked. To estimate the predictability, a quantitative measure of the difference in the descriptive blood parameter values between the study groups, the two-way Mann–Whitney rank correlation coefficient was employed, calculated using the Wendt formula. Another used coefficient was the CLES (Common Language Effect Size Statistic). Statistical significance was considered at *p* < 0.05.

## 3. Results

### 3.1. Correlations between the Values of PROMs at Successive Checkpoints

Analyzing the Spearman’s rank correlation coefficients between the results of the individual PROMs reported by patients at successive follow-up points, statistically significant correlations were observed between the results at various follow-up points. A statistically significant correlation was found between all checkpoints starting from the fourth week after PRP injection (i.e., from the second assessment) for the VAS and QDASH scales (*p* < 0.05). However, within the PRTEE scale, no statistical significance was observed between the results obtained at 24 and 104 weeks of observation. Detailed results of the the PROM correlation analysis are presented in Table 2.

### 3.2. Identification of Factors Differentiating Outcomes between the MCID+ and MCID− Groups

The conducted statistical analysis revealed significant differences between the MCID+ and MCID− groups within the VAS, QDASH, and PRTEE regarding the baseline values of parameters such as EOS%, EOS 10^9^/L, BASO%, MCV fL, MPV, MCH pg, and the MPV/PLT ratio (Table 3).

Based on the identified significant differences (Table 2), it was demonstrated that the level of individual blood morphological elements can influence the treatment outcome, measured using individual PROMs. For the VAS scale, factors favoring a good treatment outcome were higher values of EOS%, EOS 10^9^/L, and BASO%. For the QDASH scale, these were a lower value of MCV fL and a higher MCH pg. The differentiating factors for patients in the MCID+ group from those in the MCID− group for the PRTEE scale were MPV/PLT, MPV, EOS%, and EOS 10^9^/L.

### 3.3. The Evaluation of Effect Size of the Determined Level of Selected Blood Morphological Elements and the MPV/PLT Ratio on the Treatment Outcome

Next, the impact of the results for EOS%, EOS 10^9^/L, BASO%, MCV fL, and MCH pg on the MCID outcome (MCID+ and MCID−) in both groups was examined using the two-serial rank correlation and CLES. This allowed for assessing the effect of the above-mentioned significant blood morphological parameters on the affiliation of patients with the MCID+ or MCID− group depending on their values (Table 2). The highest statistically significant association between MCID+ or MCID− affiliation was observed within the VAS scale for EOS% (r_g_ = 0.58), within the QDASH scale for the level of MCV fL (r_g_ = 0.59), and within the PRTEE for the level of MPV fL (r_g_ = 0.96). CLES, on the other hand, within the VAS scale for EOS% and QDASH for MCV fL showed a substantial, in both cases, 79% difference, and PRTEE for MPV fL showed a 98% difference between the MCID+ and MCID− groups.

Finishing Section 3 it is important to add that during the administration of PRP, as well as throughout the observation, no complications were recorded, besides pain during the injection.

### 3.4. Treatment Success

According to the MCID measurements, 30% to 44% of the PRP treatments were successful, depending on the various PROMs: 31/107 elbows (30%) for QDASH, 33/107 (31%) for the VAS, and 47/107 (44%) for PRTEE tests. The results obtained by us are less promising than those reported by other authors [11,12,13,14,16,17,18,19,20,21]. In our opinion, this stems from the way patients were selected in the current study. In contrast to other authors, we recorded a good result only in patients with a sustained improvement (MDID+) at all follow-up points from the fourth week [11,12,13,14,16,17,18,19,20,21].

## 4. Discussion

One of the most serious complaints inherently associated with pathological processes, including lateral elbow tendinopathy (LET), is pain. Pain is the primary complaint of patients with LET and simultaneously one of the most important perceptible reasons for upper limb function limitation. Therefore, in the context of the current study, the most significant results are the positive impact of the EOS% and BASO% levels on the treatment outcomes measured on the VAS scale, the positive predictive effect of MCV on QDASH, and of MPV on PRTEE. The above results are even more deserving of attention because restrictive inclusion and exclusion criteria were applied, involving the selection of study participants who either achieved a sustained improvement (MCID+) from the fourth week of observation or experienced deterioration (MCID−) at all time follow-up points of the study. Despite the strict eligibility criteria for the study, the success rate of 30–44% in our study is in accordance with the publication of Mishra and Ben-Nafa [16,20].

Platelet-rich plasma (PRP) and its application remain an important topic in the discussion of treating various orthopedic conditions, including enthesopathies. Attempts to use PRP also occur in the treatment of chronic wounds and in preventing and limiting infections [24]. The effects of PRP in this regard are associated with the presence of various cells and substances in the administered material, the influence of which on the regeneration process and wound healing has been proven [25]. In the pathophysiology of the inflammatory process, eosinophils have long been perceived as intensifying its severity and participating in the inflammatory response to the penetration of parasites and contact with allergens. However, research from the last decade suggests that eosinophils should not be perceived so one-dimensionally. Currently, eosinophils are considered more to be regulators of homeostasis than just pro-inflammatory cells [26]. This has even led to the formulation of the hypothesis (the LIAR hypothesis) that eosinophils play a significant role in regulating signaling pathways in the body, not only in defense reactions but also in tissue regeneration and oncogenesis [27].

**Figure 2:**
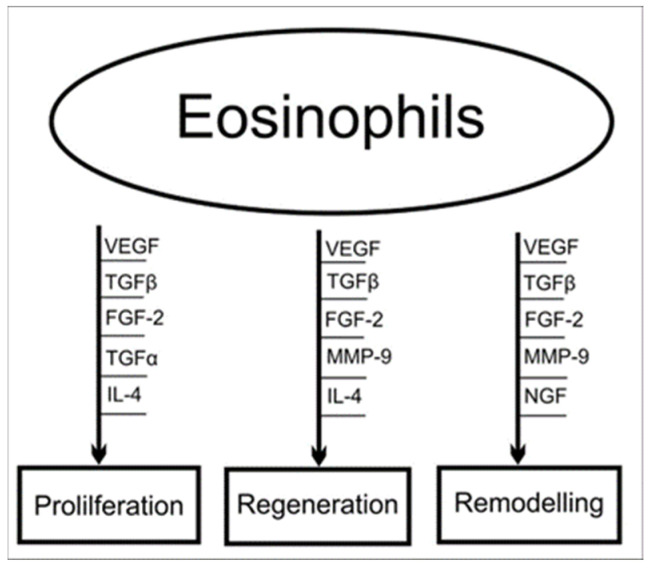
The role of eosinophils in the process of proliferation, remodeling, and tissue regeneration in tendinopathy.

The regulation and neutralization of excessive inflammatory processes is possible thanks to the substances released from the eosinophils, including tumor necrosis factor-alpha (TNF-alpha), transforming growth factor (TGF), and platelet-activating factor [28]. These mediators also participate in the processes of regeneration and remodeling, including scar formation and wound healing. In addition to secreting TGF and stimulating fibroblasts, suggested areas of eosinophil “activity” include influencing epithelial proliferation, releasing metalloproteinases, and participating in the structuring of collagen fibers [29,30]. The authenticity of these assumptions is supported by the ability of eosinophils to secrete fibroblast growth factor-2 (FGF-2), nerve growth factor (NGF), and vascular endothelial growth factor (VEGF), which play a significant role in the remodeling phase of a healing wound [28]. Ongoing studies on animal models provide a basis for considering eosinophils one of the important factors influencing both proper wound healing and the limitation of inflammatory conditions associated with tissue regeneration [31,32,33]. Furthermore, the research results indicate that eosinophils, through their reaction to interleukin-4 (IL-4) and their own secretion of it, are involved in nerve regeneration, both in spontaneous regeneration and regeneration assisted by acellular scaffolds. The role played by eosinophils in the processes described above are schematically presented in Figure 2.

However, it should be emphasized that these studies suggest that the involvement of eosinophils in nerve regeneration is significant but not crucial [34,35,36]. Basophils also show a connection with IL-4 secretion. Unlike eosinophils, however, they are believed to mainly play a secretory role, acting as a source of IL-4 and an activator of fibroblasts [37]. Studies conducted by Sicklinger et al. on a mouse model demonstrated that low levels of basophils in the blood were associated with a worse prognosis after a heart attack, described as a larger area of scarring. Researchers concluded that tissue repair in this case occurs with the involvement of basophils secreting IL-4 and interleukin-13 (IL-13) [38].

Regardless of eosinophils, blood platelets and their describing parameters are also considered to be associated with the presence and intensity of inflammation. Platelet volume indices, particularly the mean platelet volume (MPV) and its ratio to the platelet count (MPV/PLT), have gained interest in clinical observations. An increase in MPV is usually associated with a decrease in platelet count [39,40,41]. This is a result of the principles of platelet physiology. The body maintains mass, not the number of platelets [42]. Changes in the ratio of platelet volume to platelet count (MPV/PLT) may, therefore, be related to disturbances in the normal physiology of the platelets. Moreover, MPV in some inflammatory diseases may be an additional marker indicating the severity of the disease [43]. Studies conducted in patients with ulcerative colitis, appendicitis, and newborns with acute respiratory disorders have revealed a relationship between MPV and the severity of the clinical condition. However, there is no conclusion as to whether the variability in the indicator is only a useful additional diagnostic marker or just one of the parameters affected by the developing inflammatory state [44,45,46,47]. It is worth emphasizing that a higher MPV is associated with greater platelet activity [48,49,50]. Large platelets have a higher amount of alpha granules containing growth factors such as platelet-derived growth factor (PDGF), TGF-β, or VEGF [51,52]. The level of MPV also seems to be significant when comparing protocols and kits for preparing PRP. It has been shown that MPV differs between individual layers of centrifuged plasma and is such an important parameter of platelet activity that it should be used for PRP standardization [53].

The effectiveness of PRP may also be influenced by basophils, which are granulocytes that accumulate histamine in their granules and participate in allergic processes and IgE-dependent reactions. It has also been shown that basophils have the ability to produce cytokines such as interleukin-3 (IL-3), IL-4, interleukin-5 (IL-5), interleukin-6 (IL-6), and interleukin-1 (IL-1) [32], which may affect their role in tissue regeneration processes [54]. The mechanisms controlling cytokine secretion differ from those controlling histamine secretion. Calcium ions probably have the greatest impact on the secretion of IL-4 by basophils [55]. By secreting IL-4, basophils may participate in controlling the inflammatory process in damaged tissues, promoting the transformation of macrophages into the M2 profile (CD 206+). However, further research is needed to confirm the role of basophils in tissue regeneration [55].

In summary, the cells present in whole blood, including platelets, eosinophils, and basophils, appear to influence regenerative processes and, consequently, the effectiveness of PRP. Platelets are an obvious component associated with PRP efficacy, as they constitute the main ingredient of this preparation. However, it seems that not the quantity but rather the size of platelets and the ratio of size to quantity (MPV/PLT) have the primary impact on PRP’s action. Additionally, other immune system cells, such as eosinophils and basophils, typically not associated with PRP activity may also influence its effectiveness. Although their role in regenerative processes may be significant, a thorough understanding of their exact mechanisms requires further research.

According to the authors, the impact of the eosinophil concentration, basophils, as well as the value of MPV and the utility of the MPV/PLT ratio are worth further investigation and detailed analysis. Confirmation of our results in subsequent studies can certainly contribute to a more personalized decision regarding LET therapy using PRP and enhance our understanding of regenerative processes in the course of LET.

However, we are aware of the limitations of the current study, such as a relatively small study group and the absence of more objective methods for assessing treatment outcomes—ultrasound (USG) and magnetic resonance imaging (MRI). In further research, in our opinion, it would also be advisable to create a control group. Furthermore, in the current study, a cohort model was adopted; however, in the future, it is justifiable to utilize a control group, which will be helpful in confirming the results of the current analysis.

## 5. Conclusions

The conclusions from our study indicate that specific blood parameters, namely EOS, BASO, MCV, and MPV, have a noticeable impact on classifying patients into MCID+ or MCID− groups. Consequently, the analysis of fundamental morphological blood parameters, with a focus on EOS, BASO, MCV and MPV, appears promising in predicting the effectiveness of PRP therapy for individual patients and forecasting treatment success, as measured by indicators such as VAS, QDASH, and PRTEE. The outcomes of this study pave the way for a more informed and personalized approach to LET therapy using PRP in the future, emphasizing the potential of these blood parameters as valuable indicators of treatment outcomes.

## Figures and Tables

**Table 1:** Panel of laboratory tests.

Red Blood Cell and Hemoglobin Parameters	Leukocytes	Platelet Parameters
- Red Blood Cell Count (RBC) - Hematocrit (HCT) - Red Blood Cell Distribution Width (RDW) - Standard Deviation (RDW-SD) - Coefficient of Variation (RDW-CV) - Mean Corpuscular Volume (MCV) - Hemoglobin (HGB) - Mean Corpuscular Hemoglobin (MCH) - Mean Corpuscular Hemoglobin Concentration (MCHC)	- Eosinophils (EOS%) - Basophils (BASO%) - Lymphocytes (LYM%) - Monocytes (MONO%) - Neutrophils (NEU)	- Platelets (PLT) - Mean Platelet Volume (MPV) - Platelet Distribution Width (PDW)

**Table 2:** Spearman’s correlation coefficients (r_s_) for individual PROMs in the study group.

PROM	Week of Follow-Up	Spearman’s Rank Correlation Coefficients (r_s_)						
VAS		week of follow-up						
(*n* = 46)		2	4	8	12	24	52	104
	2	-	0.53	0.48	0.51	0.30	0.33	0.16 *
	4	0.53	-	0.71	0.63	0.57	0.52	0.30
	8	0.48	0.71	-	0.77	0.51	0.46	0.30
	12	0.51	0.63	0.77	-	0.55	0.51	0.38
	24	0.30	0.57	0.51	0.55	-	0.65	0.40
	52	0.33	0.52	0.46	0.51	0.65	-	0.50
	104	0.16 *	0.30	0.30	0.38	0.40	0.50	-
QDASH		week of follow-up						
(*n* = 47)		2	4	8	12	24	52	104
	2	-	0.65	0.55	0.44	0.34	0.31	0.25 *
	4	0.65	-	0.72	0.61	0.44	0.34	0.24
	8	0.55	0.72	-	0.70	0.56	0.41	0.33
	12	0.44	0.61	0.70	-	0.58	0.53	0.50
	24	0.34	0.44	0.56	0.58	-	0.58	0.49
	52	0.31	0.34	0.41	0.53	0.58	-	0.66
	104	0.25 *	0.24	0.33	0.50	0.49	0.66	-
PRTEE		week of follow-up						
(*n* = 49)		2	4	8	12	24	52	104
	2	-	0.47	0.33	0.26	0.26	0.28	0.08 *
	4	0.47	-	0.71	0.51	0.50	0.28	0.25
	8	0.33	0.71	-	0.69	0.57	0.38	0.26
	12	0.26	0.51	0.69	-	0.59	0.47	0.27
	24	0.26	0.50	0.57	0.59	-	0.56	0.20 *
	52	0.28	0.28	0.38	0.47	0.56	-	0.24
	104	0.08 *	0.25	0.26	0.27	0.20 *	0.24	-

Legend: PROM, patient-reported outcome measure; VAS, Visual Analog Scale; QDASH, Quick Version of Disabilities of the Arm, Shoulder, and Hand score; PRTEE, Patient-Rated Tennis Elbow Evaluation. * lack of statistical significance (*p* ≥ 0.050).

**Table 3:** Significant differences between the MCID+ and MCID− groups in terms of blood test results and the strength of the effect of the level of significance of blood morphotic components.

PROM	Parameter	Groups				Results		
		MCID+ (n = 33)		MCID− (n = 13)				
		Median	QD	Median	QD	*p*	r_g_	CLES
VAS	EOS%	2.50	1.50	1.50	0.25	0.003	0.58	0.79
	EOS 10^9^/L	0.17	0.09	0.09	0.04	0.045	0.38	0.69
	BASO%	0.50	0.10	0.40	0.05	0.014	0.47	0.74
QDASH	MCV fL	29.30	1.20	93.15	1.75	0.001	0.59	0.79
	MCH pg	32.80	0.50	30.40	0.95	0.039	0.37	0.68
PRTEE	MPV/PLT	0.04	0.01	0.03	0.00	0.041	0.87	0.94
	MPV fL	9.40	0.65	7.80	0.00	0.025	0.96	0.98
	EOS%	2.40	1.35	1.05	0.15	0.043	0.86	0.93
	EOS 10^9^/L	0.13	0.08	0.06	0.00	0.041	0.87	0.94

Legend: BASO, basophils; CLES, Common Language Effect Size; EOS, eosinophils; MCID, minimal clinically important difference; MCH, mean corpuscular hemoglobin; MCV, mean corpuscular volume; MPV, mean platelet volume; PLT, platelets; PROM, patient-reported outcome measure; PRTEE, Patient-Rated Tennis Elbow Evaluation; r_g_, glass rank biserial correlation coefficient (effect strength); QD, quartile deviation; QDASH, Quick Version of Disabilities of the Arm, Shoulder, and Hand score; VAS, Visual Analog Scale.

## Data Availability

Data are contained within the article.
